# Characterizing Patient-Clinician Communication in Secure Medical Messages: Retrospective Study

**DOI:** 10.2196/17273

**Published:** 2022-01-11

**Authors:** Ming Huang, Jungwei Fan, Julie Prigge, Nilay D Shah, Brian A Costello, Lixia Yao

**Affiliations:** 1 Department of Artificial Intelligence and Informatics Mayo Clinic Rochester, MN United States; 2 Center for the Science of Health Care Delivery Mayo Clinic Rochester, MN United States; 3 Center for Connected Care Mayo Clinic Rochester, MN United States; 4 Department of Quantitative Health Sciences Mayo Clinic Rochester, MN United States

**Keywords:** patient portal, secure message, patient-clinician communication, workload, response time, message round

## Abstract

**Background:**

Patient-clinician secure messaging is an important function in patient portals and enables patients and clinicians to communicate on a wide spectrum of issues in a timely manner. With its growing adoption and patient engagement, it is time to comprehensively study the secure messages and user behaviors in order to improve patient-centered care.

**Objective:**

The aim of this paper was to analyze the secure messages sent by patients and clinicians in a large multispecialty health system at Mayo Clinic, Rochester.

**Methods:**

We performed message-based, sender-based, and thread-based analyses of more than 5 million secure messages between 2010 and 2017. We summarized the message volumes, patient and clinician population sizes, message counts per patient or clinician, as well as the trends of message volumes and user counts over the years. In addition, we calculated the time distribution of clinician-sent messages to understand their workloads at different times of a day. We also analyzed the time delay in clinician responses to patient messages to assess their communication efficiency and the back-and-forth rounds to estimate the communication complexity.

**Results:**

During 2010-2017, the patient portal at Mayo Clinic, Rochester experienced a significant growth in terms of the count of patient users and the total number of secure messages sent by patients and clinicians. Three clinician categories, namely “physician—primary care,” “registered nurse—specialty,” and “physician—specialty,” bore the majority of message volume increase. The patient portal also demonstrated growing trends in message counts per patient and clinician. The “nurse practitioner or physician assistant—primary care” and “physician—primary care” categories had the heaviest per-clinician workload each year. Most messages by the clinicians were sent from 7 AM to 5 PM during a day. Yet, between 5 PM and 7 PM, the physicians sent 7.0% (95,785/1,377,006) of their daily messages, and the nurse practitioner or physician assistant sent 5.4% (22,121/408,526) of their daily messages. The clinicians replied to 72.2% (1,272,069/1,761,739) patient messages within 1 day and 90.6% (1,595,702/1,761,739) within 3 days. In 95.1% (1,499,316/1,576,205) of the message threads, the patients communicated with their clinicians back and forth for no more than 4 rounds.

**Conclusions:**

Our study found steady increases in patient adoption of the secure messaging system and the average workload per clinician over 8 years. However, most clinicians responded timely to meet the patients’ needs. Our study also revealed differential patient-clinician communication patterns across different practice roles and care settings. These findings suggest opportunities for care teams to optimize messaging tasks and to balance the workload for optimal efficiency.

## Introduction

A patient portal is a secure online platform that allows patients to conveniently access and manage personal health information and communicate with their clinicians [1]. After the Health Information Technology for Economic and Clinical Health Act of 2009, patient portals have gained widespread adoption by health care systems in the United States [2,3]. In 2017, over 90% of health care organizations including the Veterans Administration, Mass General Brigham, Kaiser Permanente, and Mayo Clinic offered patient portal access to their patients [4]. Patient portals give patients 24-7 access to their health information (eg, clinical visits, lab test results, medications, and discharge summaries) from anywhere with internet connection [5] and have been shown to improve patient self-management by promoting the awareness of disease knowledge, status, and progress [2].

A significant function in patient portals is patient-clinician secure messaging, which enables patients and clinicians to timely communicate on a wide spectrum of issues. Patients use secure messaging to request medical appointments and refill prescriptions online [6,7]. Clinicians send patients appointment reminders and promote timely preventative care [8,9]. Patients and clinicians can communicate back and forth on complex situations such as new symptoms, disease follow-ups, medication concerns, and other medical questions. Evidence suggests that secure messaging improves health care efficiency, productivity, and quality. For instance, Zhou et al [10] investigated more than 4000 users at Kaiser Permanente before and after the introduction of a secure message system and found that their annual rates of in-person primary care visits were reduced by 9.7% after their adoption of secure messaging. Simon et al [11] showed that both antidepressant adherence rate and depression treatment satisfaction increased by 20% among patients using secure messaging.

On the other hand, the secure messaging resulted in additional workload for clinicians and contributed to work burnout, as it increased patient-clinician interactions between in-person patient visits. According to a survey, 63% of 43 clinicians across 5 clinics disagreed with the notion that “secure messaging reduces my workload,” and 33% agreed with the notion that “secure messaging has a negative effect on my workflow” [12]. Another study shows that primary care physicians spend, on average, 1.4 hours of their workday (5.9 hours) interacting with electronic health records for non–face-to-face care after clinic hours [13].

With the increase in the number of patients signing up for these portals, the number of secure messages has risen substantially, especially during the COVID-19 pandemic [14-18]. It will be critical to the care teams to understand the patient-clinician messaging and to properly distribute the communication load for better efficiency and avoiding clinician burnout. It could be foreseen that some health care systems would be likely to face the challenge of managing the increasing volume of patient messages soon [12], which will require new billing models and practice metrics, or additional infrastructures, including support staffs to reply to the increasing volume of patient messages. However, there is limited understanding of the use of secure messaging and the extent of users’ interaction through this medium including clinician messaging load, messaging time delay, messaging time distribution in a day, and messaging complexity of a communication thread.

In this study, we attempted to bridge this knowledge gap by analyzing more than 5 million secure messages that were generated by patients and clinicians between 2010 and 2017 at a large multispecialty health system at Mayo Clinic, Rochester. We performed message-oriented and sender-oriented analyses by calculating the message volumes, patient or clinician population sizes, message counts per patient or clinician, time distribution of clinician messaging, and their trends over the years. We also performed thread-oriented analysis to probe the time delay in clinician responses to patient messages to assess their communication efficiency and the back-and-forth rounds to estimate the communication complexity. Our findings shed light on the patient-clinician digital communication and inform future improvement in the use of secure medical messaging.

## Methods

### Data Collection and Preprocessing

The patient portal (Patient Online Services) at Mayo Clinic, Rochester [19] was started in 2010 for primary care practice and later extended to specialty practice in 2013. The patient portal allows patients and clinicians to communicate bidirectionally via secure messaging on a wide range of issues. We retrieved more than 5 million secure messages from the patient portal between February 18, 2010, and December 31, 2017. Each message has a unique identifier (ID), previous message ID, initial message ID, sender ID, recipient ID, the timestamp when it was sent, message subject, and message body. In a message thread with a series of back-and-forth messages, the initial message is the first message initiated by a sender, and the message ID of the initial message serves as the ID of the message thread. We then applied three filters: exclude the messages with empty message bodies; exclude the messages sent by mock-up patients and clinicians that were created for testing; and exclude messages sent by a clinician group where the sender uses a shared ID, usually for impersonal communication. In the end, we obtained a total of 5,654,514 secure messages sent by both patients and clinicians for the following analysis.

### Message-Oriented and Sender-Oriented Analysis

We started by calculating the descriptive statistics for the approximately 5.6 million secure messages to probe four aspects of patient-clinician communication. (1) The total numbers (volumes) of messages sent by patients and clinicians and the overall counts of unique patient and clinician senders. We also distinguish whether a message is an initiated message from a sender (ie, a drug-related question from a patient or an appointment reminder from a clinician) or a replied message (ie, a follow-up question or clarification) in a message thread; (2) For patient-sent messages, we calculated the distribution of message counts per patient for the entire study period, the number of secure messages and the count of unique patient senders by year, and the distribution of message counts per patient each year. The patients who registered for patient portal but did not send any messages were excluded; (3) Similarly, for clinician-sent messages, we calculated the distribution of message counts per clinician for the entire study period, the number of secure messages and the count of clinician senders by year, and the distribution of message counts per clinician each year. In addition, we grouped the clinicians into 9 categories based on their practice roles (ie, physician, nurse practitioner/physician assistant [NP/PA], registered nurse [RN], and other) and care settings (ie, primary care, specialty, and other) as listed in Table 1, and measured the workload for each clinician category. The “Other—other” category refers to other supporting staffs who communicated with patients via secure messaging, such as patient appointment service specialists, social workers, and financial counselors who work outside of the primary and specialty care setting; and (4) To analyze the workload of clinicians in different times of a day, we split 24 hours into 12 time slices for each day (ie, 11 PM to 1 AM, 1 to 3 AM, 3 to 5 AM, 5 to 7 AM, 7 to 9 AM, 9 to 11 AM, 11 AM to 1 PM, 1 to 3 PM, 3 to 5 PM, 5 to 7 PM, 7 to 9 PM, and 9 to 11 PM) and calculated the percentage of secure messages that those clinicians sent in each of the 12 time slices by year.

**Table 1:** Clinician categories based on their practice roles and care settings.

Role	Primary care	Specialty	Other
Physician	Physician—primary care	Physician—specialty	N/A^a^
NP^b^/PA^c^	NP/PA—primary care	NP/PA—specialty	N/A
RN^d^	RN—primary care	RN—specialty	N/A
Other	Other—primary care	Other—specialty	Other—other

^a^N/A: not applicable.

^b^NP: nurse practitioner.

^c^PA: physician assistant.

^d^RN: registered nurse.

### Thread-Oriented Analysis

We investigated two aspects of patient-clinician communication within message threads. The first aspect is the time delay of clinician responses to patient messages. The analysis of time delay between patient messages and clinician responses may suggest how promptly clinicians responded to patients. We identified all the pairs of patient-sent messages and clinician-replied messages in a message thread and calculated the time difference (in days) between them. We then calculated the distribution of time delays for the entire study period and for each year. Secondly, patients often communicate with clinicians back and forth for multiple times in a message thread. We examined these message threads by measuring the number of back-and-forth rounds in each message thread (ie, length of a message thread) and calculating the distribution of message threads in terms of message thread length over time.

We developed a series of scripts in Python (Python Software Foundation) together with popular Python libraries (eg, pandas [20], NumPy [21], SciPy [22], and Matplotlib [23]) to perform data collection and preprocessing, statistical analysis, and visualization. No patients were exposed to any intervention. We used the data from the Mayo Clinic Unified Data Platform for analysis. The study was approved by the Mayo Clinic Institutional Review Board (19-002211).

## Results

### Message-Oriented and Sender-Oriented Analysis

#### Descriptive Statistics of Secure Medical Messages and Their Senders

Table 2 lists the total numbers of secure messages sent (ie, initiated and replied) by patients and clinicians as well as unique patient senders and clinician senders. The number of messages initiated by clinicians was almost identical to that of messages replied by clinicians but was slightly more than that of messages initiated by patients (1.7 million versus 1.5 million). However, the number of patient-initiated messages was about 3 times that of patient-replied messages (1.5 million versus 0.5 million). In addition, 93.7% of the patients (203,166/216,740) had initiated secure messages, whereas only 52.6% (113,974/216,740) had replied to their clinicians. This suggests that the patients in general initiated a message when they had health-related issues, rather than replying in a message thread, whereas the clinicians were obligated to initiate messages and respond to patient messages.

**Table 2:** Descriptive statistics of secure medical messages and their senders for the 8-year period.

Senders and secure message types			Numbers of secure messages, n (%)		Counts of unique senders, n (%)
**Patient**					
	Initiated messages	1,569,172 (74.06)		203,166 (93.74)	
	Replied messages	549,601 (25.94)		113,974 (52.59)	
	Total	2,118,773		216,740	
**Clinician**					
	Initiated messages	1,774,000 (50.17)		5690 (67.27)	
	Replied messages	1,761,741 (49.83)		8,070 (95.40)	
	Total	3,535,741		8459	

The ratio of overall unique patient count to overall unique clinician count was about 25:1 (216,740 versus 8450). The ratio of patient count and clinician count per year increased from 7.7 (1919 versus 249) in 2010 to 21.0 (125,647 versus 5980) in 2017 as shown in Table S1 (Multimedia Appendix 1). This indicates that the workload per clinician had an increasing trend over years in terms of patient users on the secure messaging system.

#### Patient-Sent Secure Messages

**Figure 1:**
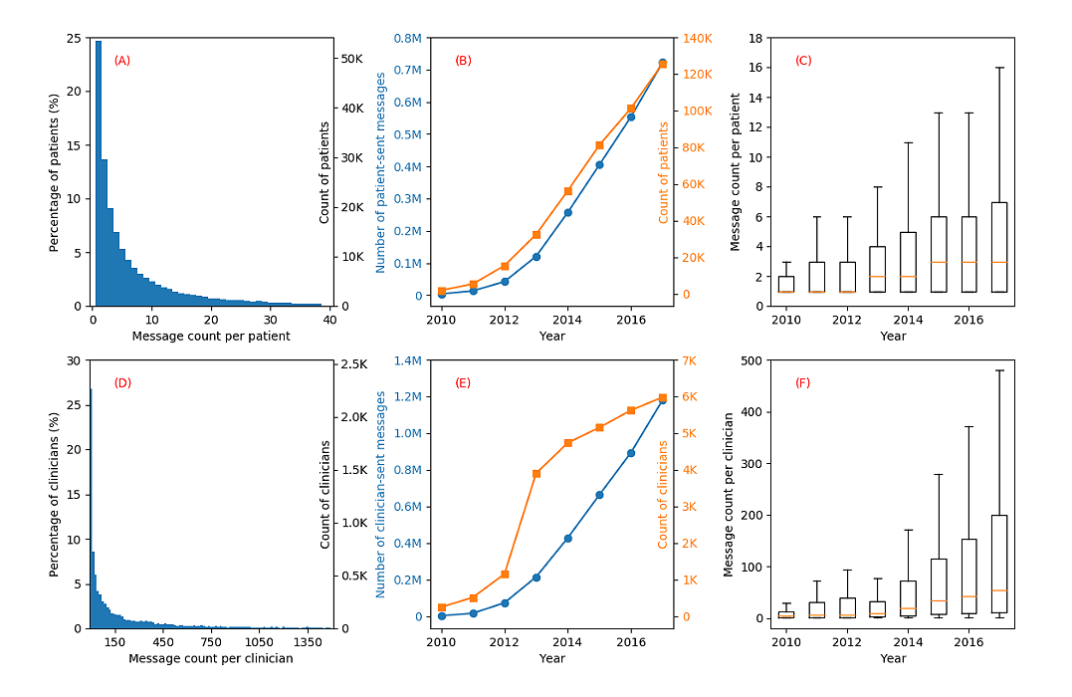
Distribution of message count per patient or clinician (A and D), total number of patient-sent or clinician-sent messages and count of unique patients or clinicians by year (B and E), and box-whisker graph of message count per patient or clinician by year (C and F).

We illustrated the distribution of message count per patient, the total numbers of patient-sent messages and unique patients by year, and the box-whisker graph of message count per patient by year in Figure 1 (A-C). We found that 95.4% (206,818/216,740) of the patients sent less than 40 messages, as shown in Figure 1 (A). The median number of patient-sent messages was 4. The maximum number of patient-initiated and patient-replied messages was 2052 and 302, respectively (Table S1, Multimedia Appendix 1). We observed a similar increasing trend between the numbers of unique patients and patient-sent messages over years, as shown in Figure 1 (B), indicating the strong adoption of this technology. The Pearson correlation coefficient between the numbers of unique patients and patient-sent messages calculated with SciPy is *r*=1. The median of message count per patient increased from 1 to 3 during 2010-2017 as depicted in Figure 1 (C).

#### Clinician-Sent Messages

Figure 1 (D-F) depicts the distribution of message count per clinician, the total numbers of clinician-sent messages and unique clinicians by year, and the box-whisker graph of message count per clinician by year. In Figure 1 (D), the distribution of message count per clinician exhibits a “long tail” pattern, where the median is 77 but the max stretches far right at 18,314 (way beyond the X axis labeling range). Both the numbers of clinicians and clinician-sent messages were also increasing over time with the growths of the numbers of patients and patient-sent messages (Figure 1 [E]). The Pearson correlation coefficient between the total numbers of clinician-sent messages and patient-sent messages over years is *r*=1, suggesting that the total number of clinician-sent message is strongly associated with that of patient-sent message. The median and IQR of message count per clinician steadily increased during 2010-2017 (Figure 1 [F]). We observed that the count of clinicians largely increased but the median and IQR of message count per clinician decreased in 2013 compared with 2012 because many specialties at Mayo Clinic, Rochester started to use the patient portal.

Between 2010 and 2017, 19.93% (6773/8459) of the clinicians transitioned out of Mayo Clinic, Rochester and, therefore, we do not have information on their practice roles and care settings. We grouped the remaining 6773 clinicians, who generated 86.87% (3,071,529/3,535,741) messages, into 9 clinician categories. Based on these messages, we then analyzed the workload, message counts per clinician, and the distribution of messaging time in a day for each clinician category.

**Figure 2:**
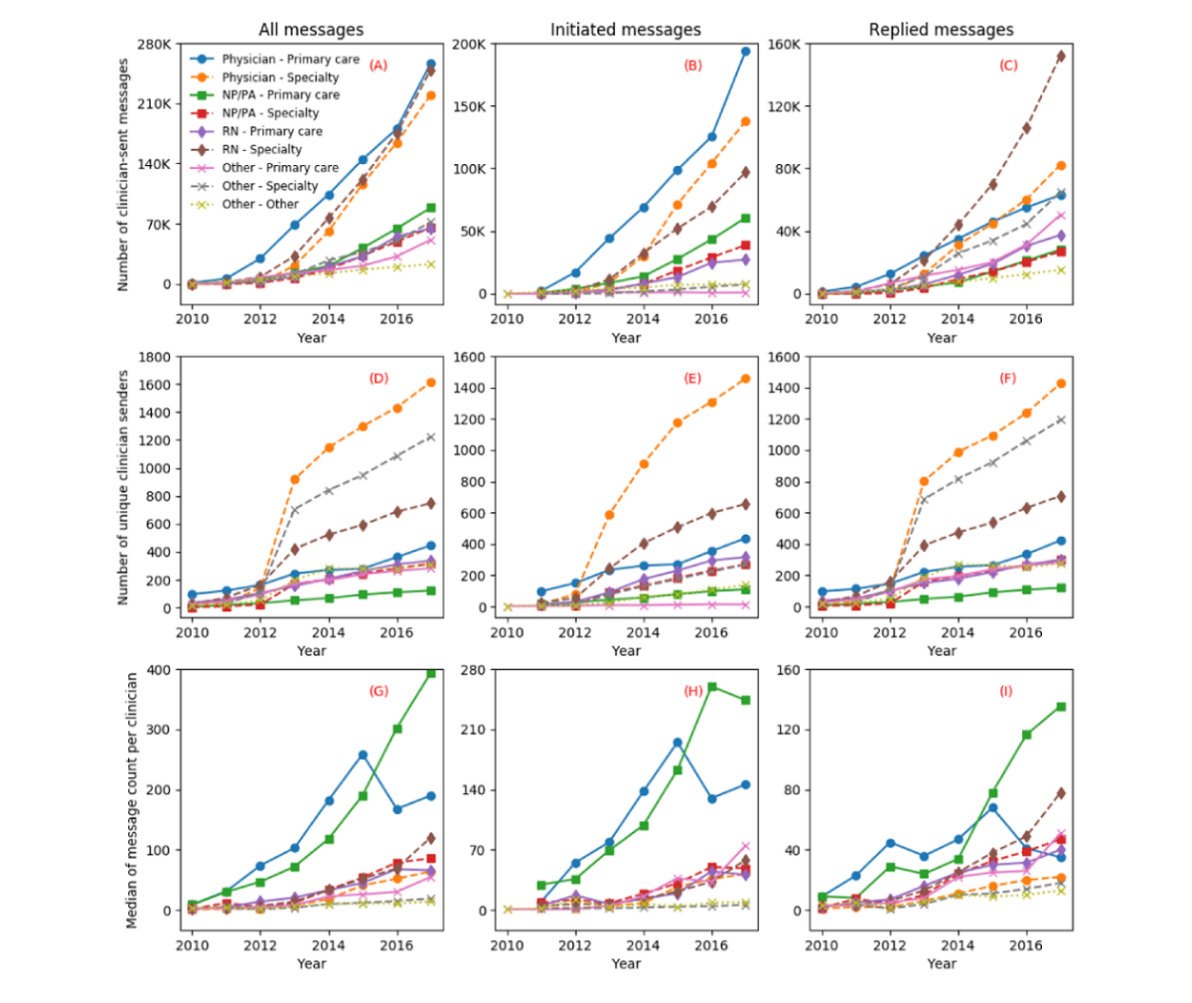
Total number of clinician-sent messages (A-C), the number of unique clinician senders (D-F), and median of message count per clinician (G-I) in each clinician category over years. NP: nurse practitioner; PA: physician assistant; RN: registered nurse.

Figure 2 shows the total number of clinician-sent messages, the total number of clinicians, and the median of message count per clinician in each clinician category for each year. As shown in Figure 2 (A), the number of messages sent by clinicians in each clinician category was progressively increasing over time. Three clinician categories, namely “physician—primary care,” “Registered Nurse (RN)—specialty,” and “physician—specialty” had the largest increase in the generated messages. After 2014, the number of messages sent by these 3 clinician categories were over 2.5 times more than those of the other clinician categories combined. The category “physician—primary care” initiated the largest number of messages every year (Figure 2 [B]). During 2010-2013, the “physician—primary care” category also replied the most to the messages from patients, but after 2013, “RN—specialty” took over the top spot in responding to patients (Figure 2 [C]).

The total numbers of unique clinician senders in each category had a steady increase during 2010-2017 (Figure 2 [D-F]). After 2012, the clinician categories with top 3 message senders were “physician—specialty,” “other—specialty,” and “RN—specialty.” Most of the clinicians in the “other—specialty” category were responsible for responding to patient messages rather than initiating messages. The “nurse practitioner (NP) or physician assistant (PA)—primary care” category has the smallest size of clinician senders (Figure 2 [D]).

In Figure 2 (G), the median of message count per clinician in 7 out of the 9 clinician categories steadily increased during 2010-2017. Among them, the top 2 clinician categories were “NP/PA—primary care” and “physician—primary care.” In 2017, the median of message count per clinician by the “NP/PA—primary care” category was over 2 times of any other groups (Figure 2 [G-I]).

#### Clinician Messaging Workload Within a Day

**Figure 3:**
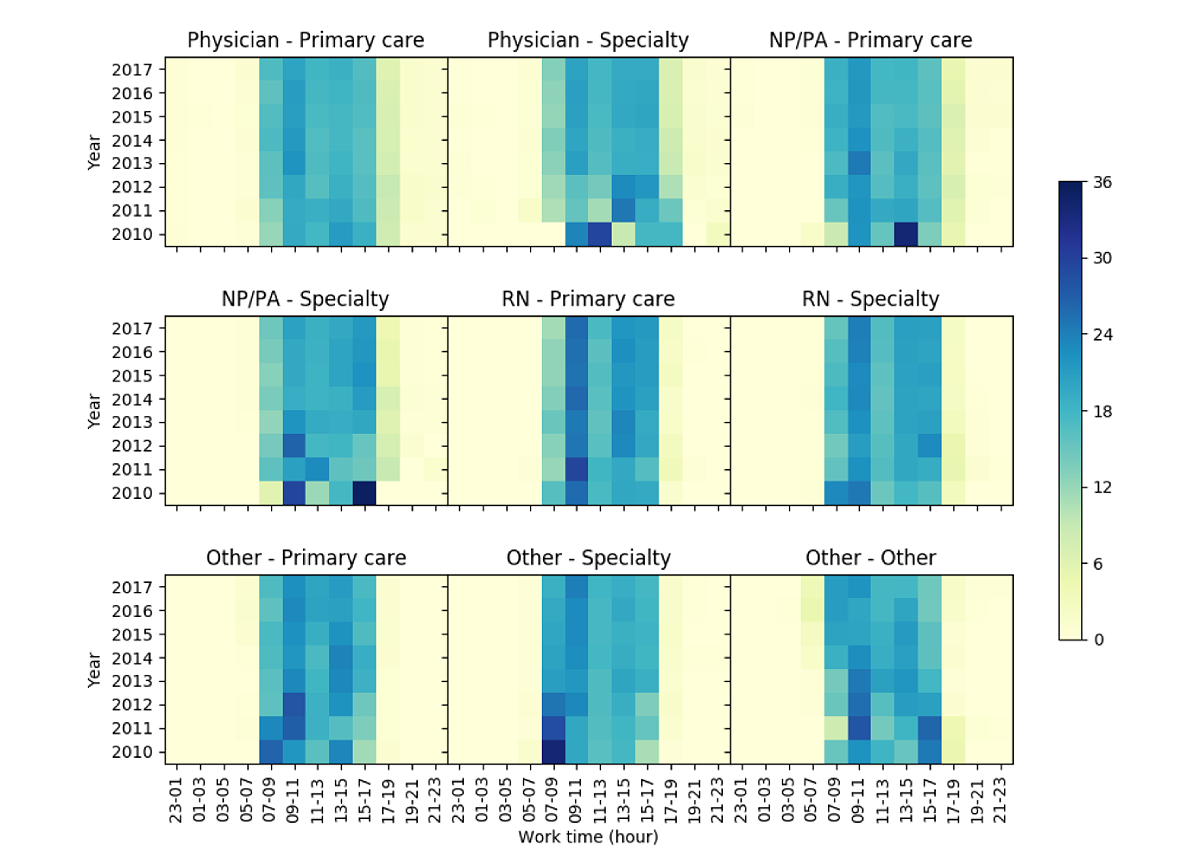
Distribution of clinician messaging time in a day. Each color block represents the percentage of messages sent in the corresponding time slice during a day. NP: nurse practitioner; PA: physician assistant; RN: registered nurse.

We analyzed the clinician messaging time during a day across the 9 clinician categories in terms of message percentage (Figure 3) and message number (Figure S5-7, Multimedia Appendix 1). Most of the messages were sent from 7 AM to 5 PM. However, physicians and NP/PA also sent a considerable number of messages to patients between 5 PM and 7 PM. For example, the percentage of messages sent by “physician—primary care” during 5-7 PM increased from 7.95% (102/1283) in 2010 to 8.98% (2669/29,705) in 2012 and then dropped to 6.12% (15,714/256,761) in 2017. The percentage of messages sent by “NP/PA—primary care” during 5-7 PM remained constant around 5.08-6.75% (3/59-2,824/41,833), between 2010 and 2017.

### Thread-Oriented Analysis

We identified 1,576,205 message threads in 3,887,542 messages, which include 1,332,931 patient-initiated messages and 243,274 clinician-initiated messages, for thread-oriented analysis. Among these message threads, we identified 1,761,739 clinician responses to patient messages.

#### Time Delay Between Patient Messages and Clinician Responses

**Figure 4:**
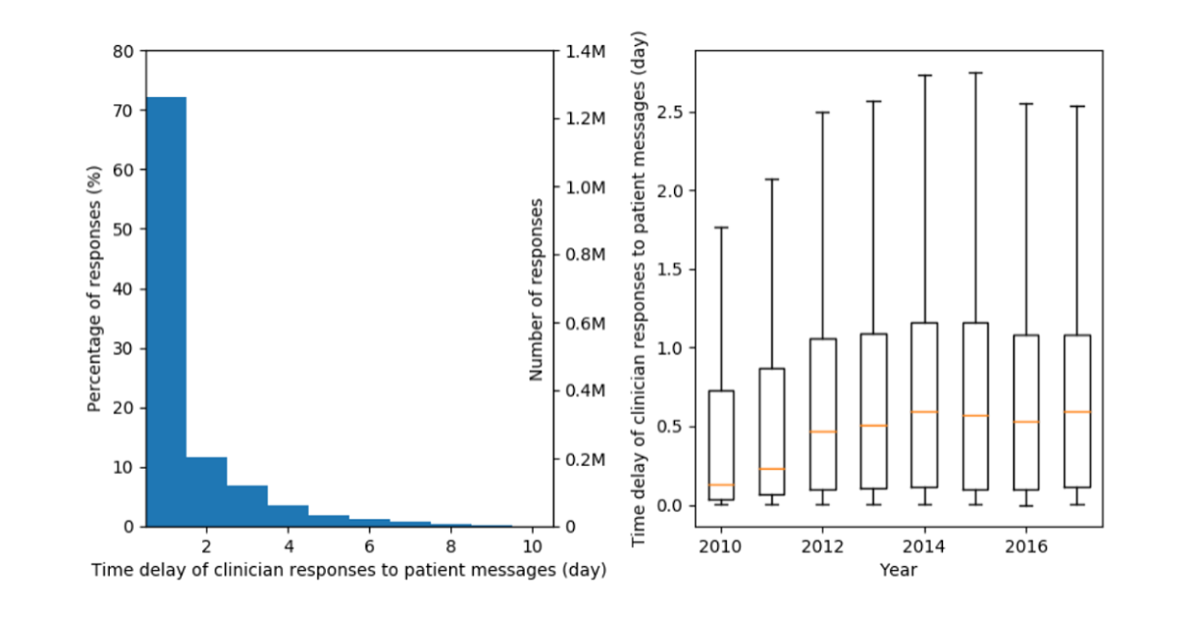
Distribution (left) and box-whisker graph (right) of time delay in the clinician responses to patient messages.

We illustrated the distribution of time delay between clinician responses and patient messages in Figure 4. It appears that 96.0% (1,691,733/1,761,739) of patient messages were responded to by clinicians within 5 days. The shortest time a clinician spent responding to patients was 1 second, and the median was 0.6 days. With the increase in the total number of clinician messages and message count per clinician, the median time delay between clinician responses and patient messages increased from 0.13 days in 2010 to 0.59 days in 2014 but remained steady (0.53-0.59 days) after 2014. The IQR of time delay showed a trend similar to the median time delay over years.

#### Back-and-Forth Messages Between Patients and Clinicians in a Message Thread

**Figure 5:**
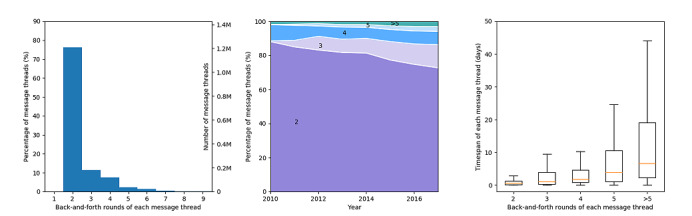
Distribution (left), percentage (middle), and timespan (right) of message threads in terms of back-and-forth rounds in the message threads.

We analyzed the distribution of message thread lengths (Figure 5). We found that 95.1% (1,499,316/1,576,205) message threads had fewer than 5 back-and-forth messages. The median and maximum lengths of the message threads were 2 and 34, respectively. Between 2010 and 2017, the percentage of message threads with a length of 2 decreased from 88.0% (3429/3895) to 72.7% (372,424/512,395). The percentage of message threads with a length of 3 increased from 0.4% (16/3895) to 13.6% (69,754/512,395). The percentage of message threads with a length of 4 remained relatively stable, 6.1-9.6% (2354/38,537-374/3895). The percentage of the message threads with a length of 5 or more showed an uptrend over time. The median timespan of message threads was elongated as the message thread lengths increased.

## Discussion

### Principal Findings

Secure messaging in patient portal provides patients more convenient access for more personalized medical advice and information [5,24]. Patients can send secure messages to their clinicians anywhere and anytime as they prefer, which fulfills a growing consumer model of medical care [14]. Similar to previous results on secure messaging [14-17], our patient portal experienced a large increase in the numbers of patients, clinicians, and secure messages during 2010-2017. We observed a strong correlation between patient count and patient message count. The growth of patients and messages per patient both contributes to the upsurge of patient messages, suggesting a strong adoption of this health care technique and a growing pattern of patient engagement. The significant volume difference between patient-initiated messages and patient-replied messages (1.5 million vs 0.5 million) indicates the behavioral variation of patients for initiating and replying to messages; in general, the patients initiated a message when they encountered health issues instead of following an existing message thread. This implies potential opportunities to engage patients in communication and increase responses in pertinent contexts.

The clinician message volume is strongly associated with patient message volume because the clinicians were obligated to communicate with the patients for providing health care support. There were also practice-specific and role-specific patterns for the clinicians. For example, “physician—primary care” initiated the largest number of messages to patients every year, indicating their major role in patient engagement. After 2013, “RN—specialty” replied the most of messages to patients, suggesting that the patient portal opened up a longed-for channel of taking patient information needs in specialty care [25], and that specialty RNs represented the first-line taskforce to accommodate the needs.

The patient portal also manifested upward trends in the ratio of patient count to clinician count and numbers of messages per patient and clinician. The findings suggest an inflation of clinician workload in the secure messaging. Specifically, the “physician—primary care” and “NP/PA—primary care” categories had the heaviest per-clinician messaging workload every year because the “physician—primary care” category generated the largest number of clinician messages and the “NP/PA—primary care” category has the smallest size of clinician senders. This indicates where optimization is most needed in easing the clinician workload. Most (93.2%, 2,862,978/3,071,529) of the clinician messages were sent from 7 AM to 5 PM but 17.3% (532,894/3,071,529) of the messages were sent by clinicians to patients around noon (11 AM to 1 PM), and 5.94% (182,468/3,071,529) were sent after 5 PM (5-11 PM). Our findings were consistent with those from previous studies that secure messaging has a negative effect on clinician workload [12], and that about 24% of the work is carried out by clinicians on electronic health records for non–face-to-face care after clinician hours [13].

Most of the clinicians responded to the patients in a timely manner. The median time delay of clinician responses to patient message remained constant (less than 0.60 days). Although the message volume and clinician workload persistently increased over years, the clinicians managed to respond to the patients in time. Regarding the message threads, the patients usually communicated with the clinicians in a few back-and-forth rounds. Possibly, patients and clinicians communicated via secure messaging for noncomplicated scenarios, or clinicians made a timely decision on a certain back-and-forth round about what acute issues and complex situations will require face-to-face visit or follow-up. The percentage of message threads with more than 2 rounds was rising, which may imply that the patients and clinicians were increasingly comfortable to communicate about more complex situations. However, further investigation is necessary to understand the potential mechanism of the messaging complexity of a communication thread.

The growing portal messages had different indications or impacts on its stakeholders. For the patients, a majority of them in this study received timely response to their concerns and requests. In the future, the patient engagement would probably continue to increase in terms of patient count and message count per patient, in particular, during and after the COVID-19 pandemic. For the clinicians, most of them think that secure messaging can have a positive effect on quality of care and patient safety, but secure messaging increased their burden of indirect patient care, as noted by Hoonakker et al [12]. In some cases, clinicians need to send messages to patients after 5 PM in order to respond to them in time. With the rise of patient engagement and message volume over time [14-17], it will be critical to properly distribute the communication load for better efficiency and for avoiding clinician burnouts. For the health care system, patient portals and secure messaging may have a favorable impact on the cost-effectiveness of care [26]. They could help to not only cut the administrative cost by alleviating the operational burden [27], but also reduce patients’ health care utilization by improving their functional status [28]. However, the detailed economical or cost-effectiveness analysis is beyond the scope of this paper, due to the difficulty of collecting relevant data. In the near future, some other health care systems will probably confront the management challenge of the fast-growing volume of patient messages [12]. These health care systems would require new policies, billing models, or additional infrastructures. For example, more NP/PA, RN, and other support staff would be involved in replying to the increasing volume of patient messages. The artificial intelligence (AI) and natural language processing (NLP) tools would even be invested and developed to support the care teams for secure messaging [29-31].

Our study has several limitations. First, the secure messages were collected from Mayo Clinic, Rochester, a tertiary care institution for complex medical conditions, and might not be representative of different patient populations or clinical settings in other parts of the country. Second, the patient secure messages after 2017 were not included in this study due to the upgrade of the patient portal system at Mayo Clinic in 2018. The COVID-19 pandemic is transforming the health care delivery via telehealth, and we would investigate the secure messaging after 2017 and the impact of the COVID-19 on the patient portal system for a future study. Third, the 9 clinician categories we used were not always accurate because a small portion of clinicians changed their practice roles and care settings during the study period. Fourth, there was no guarantee that the clinician-initiated or clinician-replied messages were always written by themselves. Finally, linking patient secure messages to other patient medical records for a more detailed study of the needs of different patient populations is out of the scope of this study, but represents an important area we would like to explore in the future.

### Conclusions

We performed message-oriented, sender-oriented, and thread-oriented analyses to probe and characterize millions of secure medical messages generated by patients and clinicians with diverse backgrounds. We analyzed the message volumes, patient or clinician population sizes, message counts per patient or clinician, and their trends over years. We computed the time distribution of clinician messaging to further understand their workload in different time slices of a day. For each message thread, we calculated the time delay between patient messages and clinician responses to examine the responding efficiency and the number of back-and-forth rounds to roughly assess the communication complexity.

Our study shows a steady rise in patient involvement, through the use of secure messaging, and workload per clinician over years. However, most clinicians were responding to the patients in a timely manner in order to meet their needs. Our findings shed light on opportunities for care teams to improve messaging tasks and optimize clinician workload and for the experts in AI and NLP to develop robust and intelligent messaging tools to support the care teams for better communication efficiency and quality. These findings offer valuable information on the digital interaction between patients and clinicians and may serve as a reference for promoting patient-centered care.

In the future, we will perform a content analysis of patient secure messages using AI and NLP and examine the patient populations in terms of socioeconomic factors by linking them to patient medical records. We will also perform a comparative study between patient portal messages and traditional health care services and a survey study to understand patient and clinician experiences on using patient portal messaging.

## Appendix

Multimedia Appendix 1 Supplementary materials.

## Abbreviations

AI: artificial intelligence
ID: identifier
NLP: natural language processing
NP: nurse practitioner
PA: physician assistant
RN: registered nurse
