# Supplementary material for: Characterizing Patient-Clinician Communication in Secure Medical Messages: Retrospective Study
Source: J Med Internet Res. 2022 Jan 11;24(1):e17273. doi: 10.2196/17273 (PMC8790696; doi:10.2196/17273)
Supplement: Multimedia Appendix 1 [file jmir_v24i1e17273_app1.docx]

Characterizing Patient-Clinician Communication in Secure Medical Messages: Retrospective Study

**Multimedia Appendix 1**

Ming Huang^1^, Jungwei Fan^1,2^, Julie E. Prigge^3^, Nilay D. Shah^2,4^, Brian A. Costello^3^, Lixia Yao^1*^

1. Department of Artificial Intelligence and Informatics, Mayo Clinic, Rochester, MN, United States
2. Center for the Science of Health Care Delivery, Mayo Clinic, Rochester, MN, United States
3. Center for Connected Care, Mayo Clinic, Rochester, MN, United States
4. Department of Quantitative Health Sciences, Mayo Clinic, Rochester, MN, United States

**Corresponding Author:**

Lixia Yao, PhD

Department of Artificial Intelligence and Informatics

Mayo Clinic

200 First Street SW

Rochester, MN, 55905

United States

Email: [lixia.cn.yao@gmail.com](mailto:lixia.cn.yao@gmail.com)

1. **Message- and sender-oriented analysis**
   1. **Descriptive statistics of medical secure messages and their senders**

**Table S1** Numbers of patient/clinician-generated messages and unique patient/clinician senders per year

| Year | Generated message | | | Unique sender | | |
| --- | --- | --- | --- | --- | --- | --- |
|  | Patient | Clinician | Clinician/Patient ratio | Patient | Clinician* | Patient/Clinician ratio |
| 2010 | 4,437 | 4,577 | 1.03 | 1,919 | 249 | 7.71 |
| 2011 | 13,734 | 19,296 | 1.40 | 5,398 | 515 | 10.48 |
| 2012 | 42,473 | 75,536 | 1.78 | 15,440 | 1,154 | 13.38 |
| 2013 | 121,017 | 220,357 | 1.82 | 32,445 | 3,900 | 8.32 |
| 2014 | 256,876 | 436,046 | 1.70 | 56,243 | 4,733 | 11.88 |
| 2015 | 404,518 | 674,607 | 1.67 | 81,183 | 5,152 | 15.76 |
| 2016 | 553,578 | 910,531 | 1.64 | 101,295 | 5,622 | 18.02 |
| 2017 | 722,140 | 1,194,791 | 1.65 | 125,647 | 5,980 | 21.01 |

*The senders of 1.83% (64,574 out of 3,535,741) clinician messages were not indicated.

- 1. **Patient-sent secure messages**

Table S2 Statistics of message count per patient

| Patient message type | Minimum | Maximum | Mean | Median |
| --- | --- | --- | --- | --- |
| Initiated messages | 1 | 2,052 | 7.7 | 3 |
| Replied messages | 1 | 302 | 4.8 | 2 |
| Total patient-sent messages | 1 | 2,072 | 9.8 | 4 |


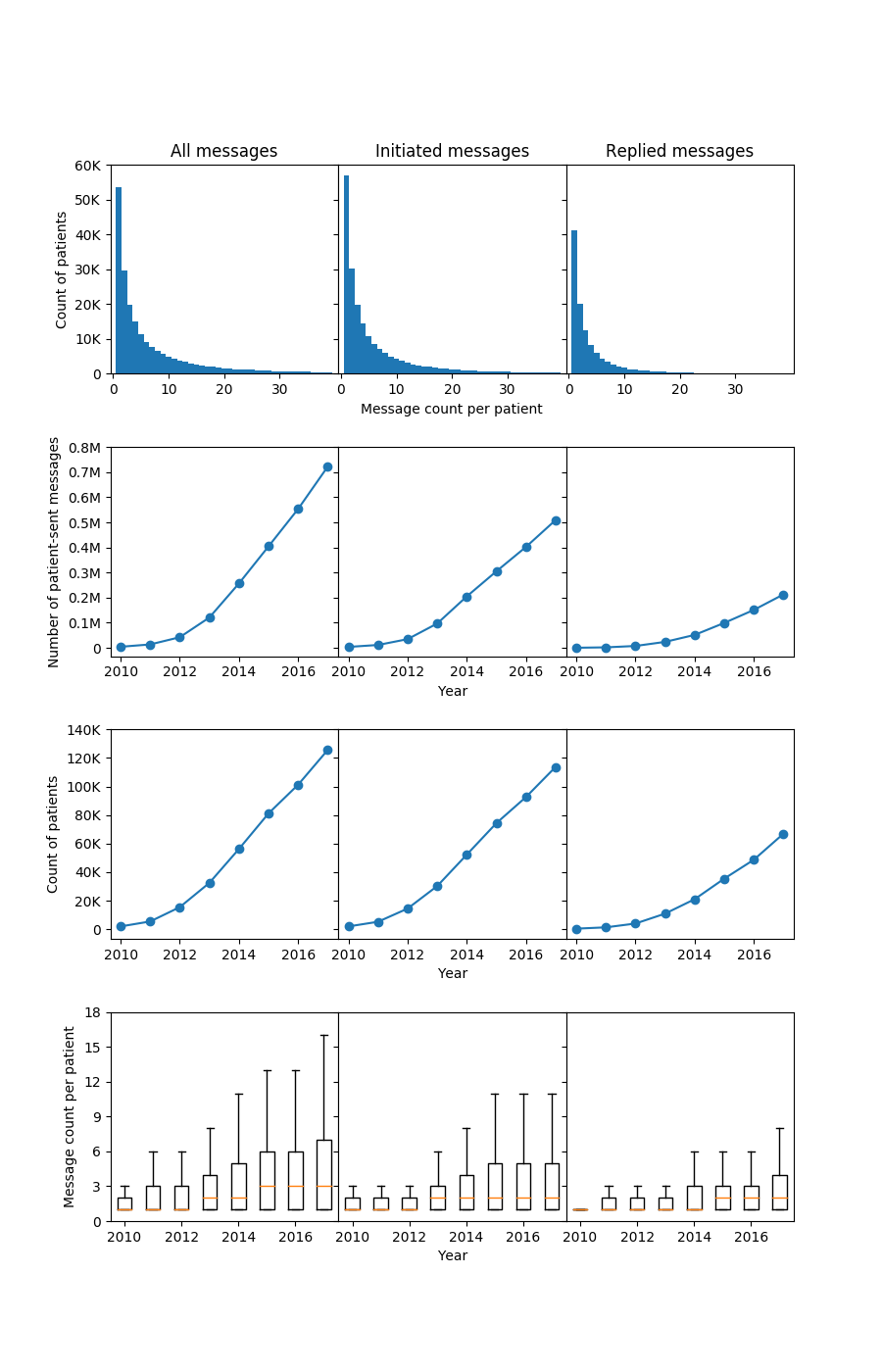


Figure S1 Distribution of message count per patient (1^st^ row, where message count is cut to 40), the number of patient-sent messages by year (2^nd^ row), the count of patients by year (3^rd^ row), and the box-whisker graph of message count per patient by year (4^th^ row)

- 1. **Clinician-sent messages**

1. Table S3 Statistics of message count per clinician

| Clinician message type | Minimum | Maximum | Mean | Median |
| --- | --- | --- | --- | --- |
| Initiated messages | 1 | 13,107 | 311.8 | 59 |
| Replied messages | 1 | 10,982 | 210.3 | 41 |
| Total clinician-sent messages | 1 | 18,314 | 410.4 | 77 |


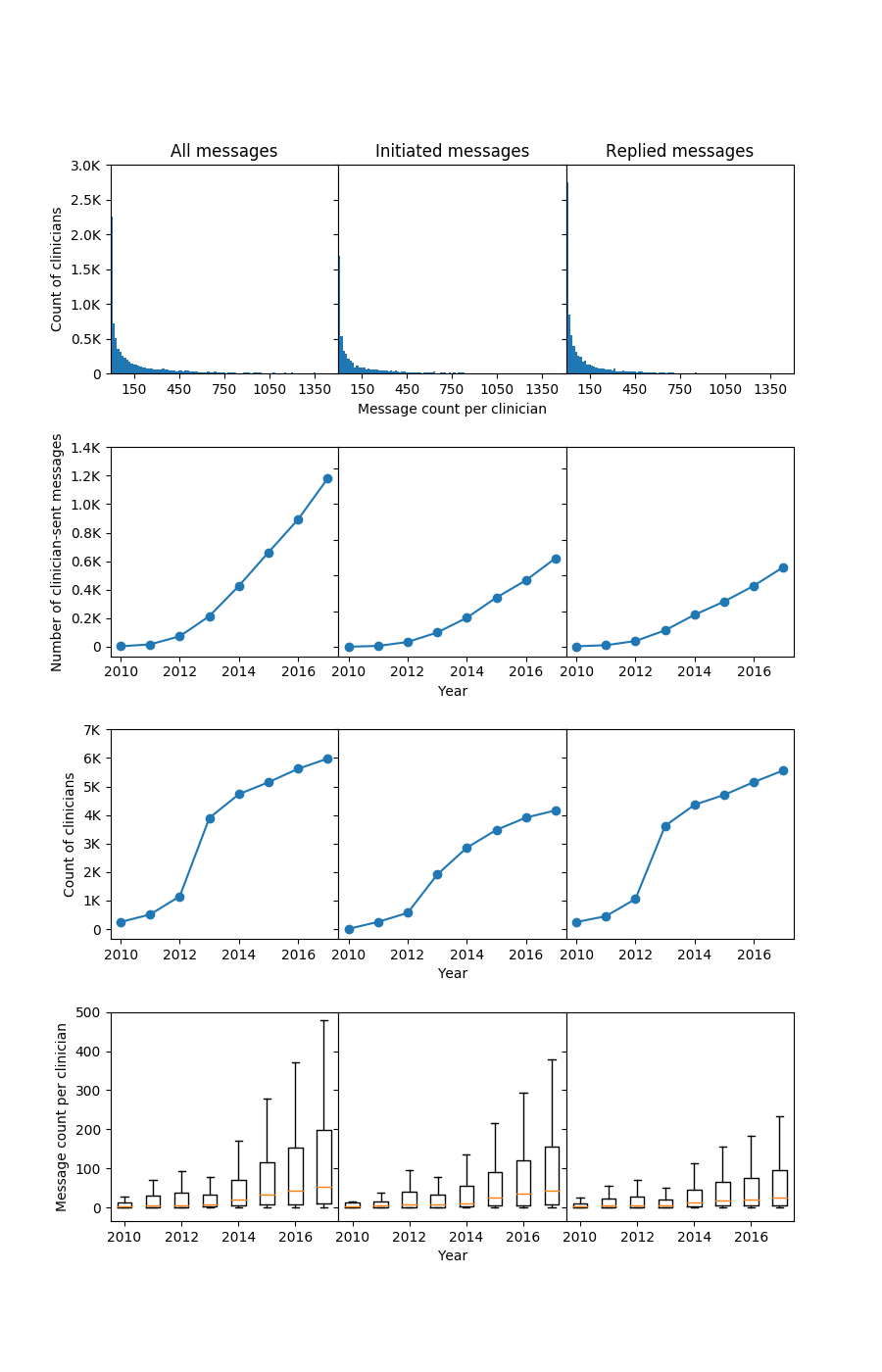


Figure S2 Distribution of message count per clinician (1^st^ row, where message count is cut to 1500), the number of clinician-sent messages by year (2^nd^ row), the count of clinicians by year (3^rd^ row), and the box-whisker graph of message count per clinicians by year (4^th^ row)

Between 2010 and 2017, 19.93% out of 8459 clinicians transitioned out of Mayo Clinic – Rochester and therefore we do not have information on their practice roles and care settings. We grouped the remaining 6773 clinicians, who generated 86.87% of 3,535,741 clinician-sent messages, into 9 clinician categories. Based on the 3.5M messages, we then analyzed the workload per clinician category, message counts per clinician, and percentages of messages sent in different time slices of a day over years.


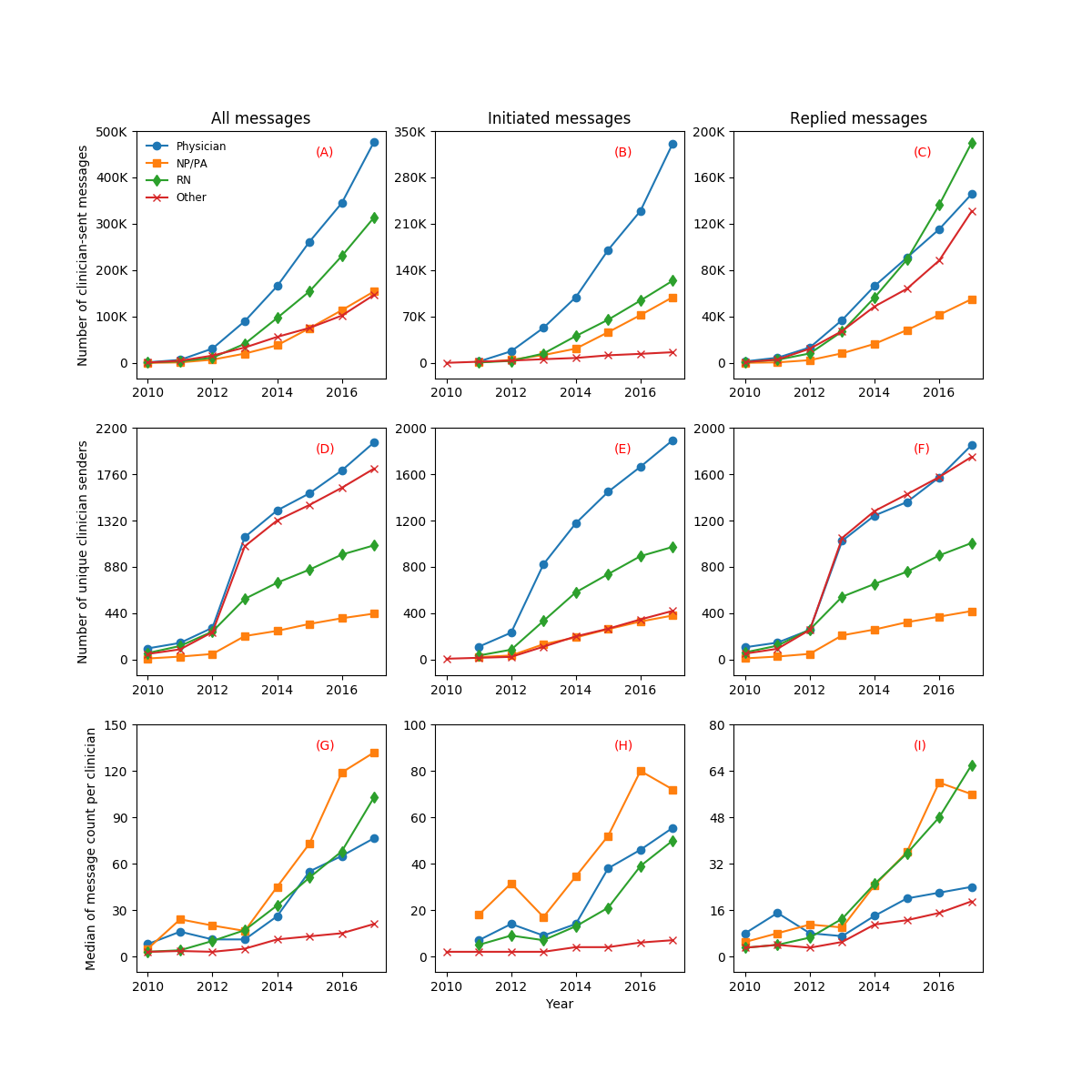


Figure S3 Total number of clinician-sent messages (A-C), number of unique clinician senders (D-F), and median of message count per clinician (G-I) in different practice roles (Physician, NP/PA, RN, or Other) over years


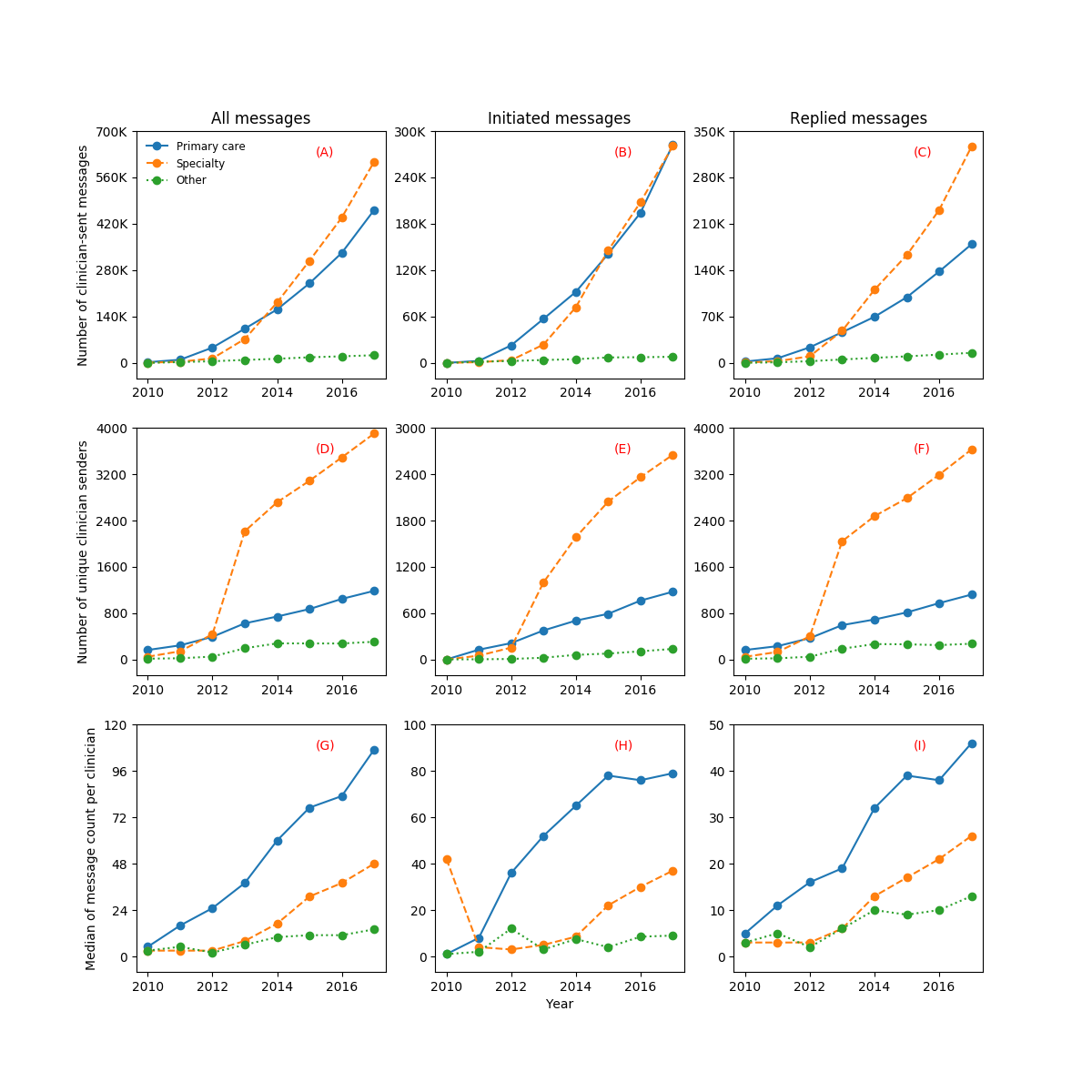


Figure S4 Total number of clinician-sent messages (A-C), number of unique clinician senders (D-F), and median of message count per clinician (G-I) in different care settings (Primary care, Specialty, and Other) over years

- 1. **Clinician messaging workload within a day**


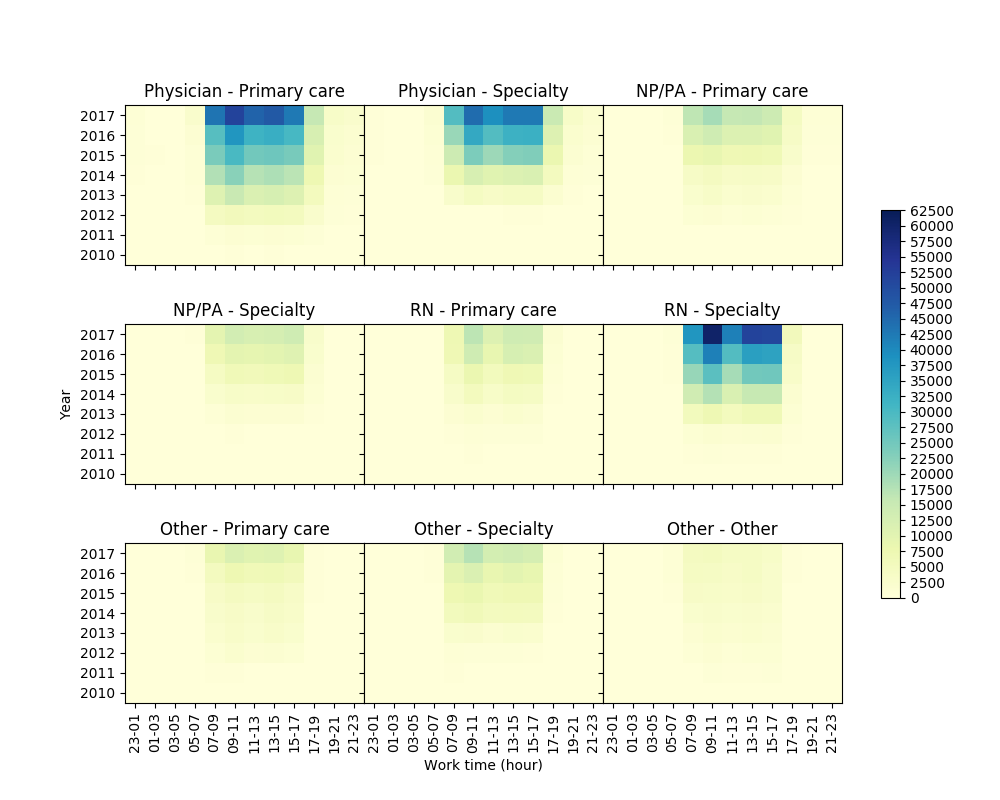


Figure S5 Number of clinician-generated messages in 12 time slices of each day over years


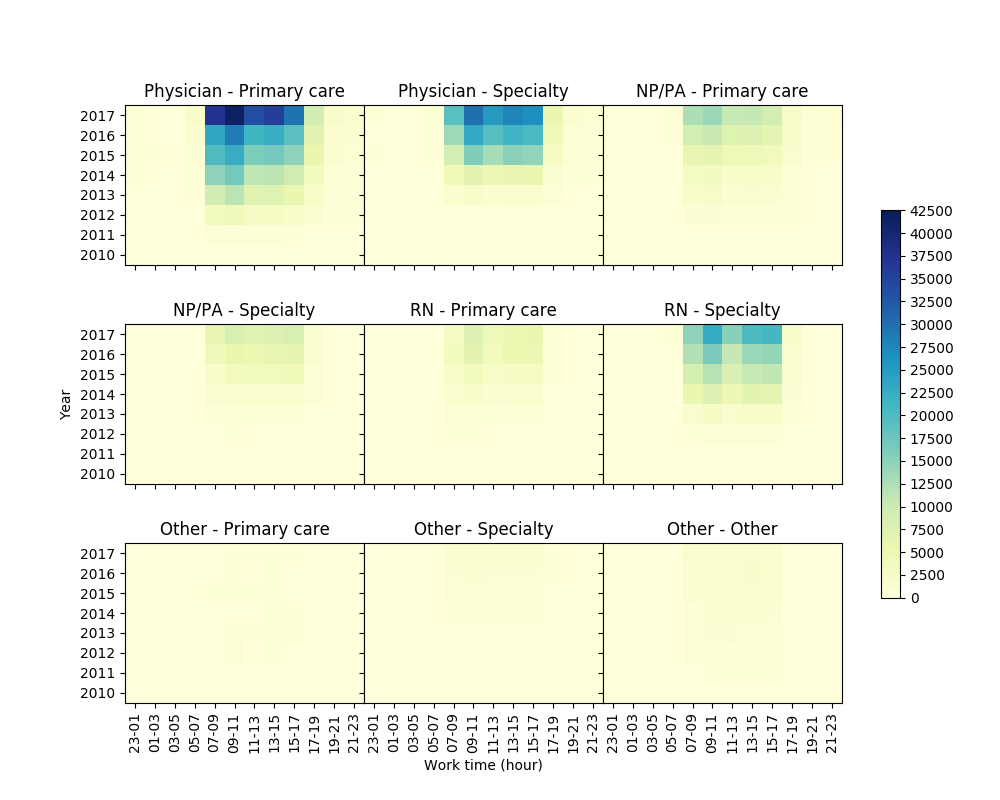


Figure S6 Number of clinician-initiated messages in 12 time slices of a day over years


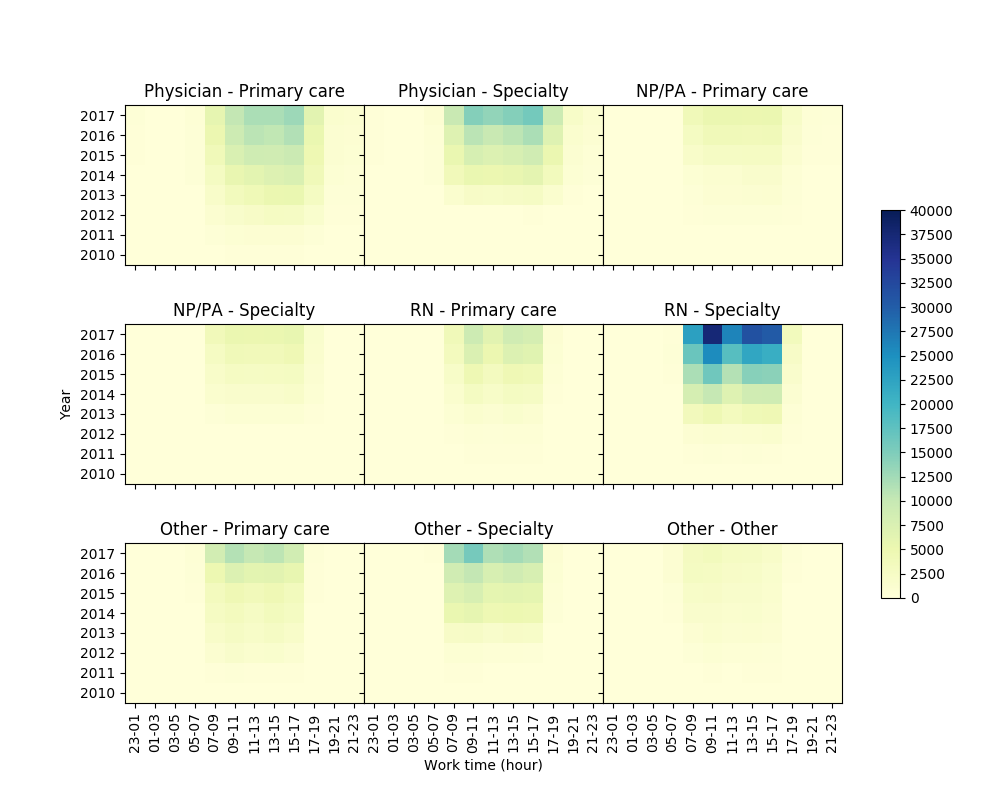


Figure S7 Number of clinician-replied messages in 12 time slices of a day over years


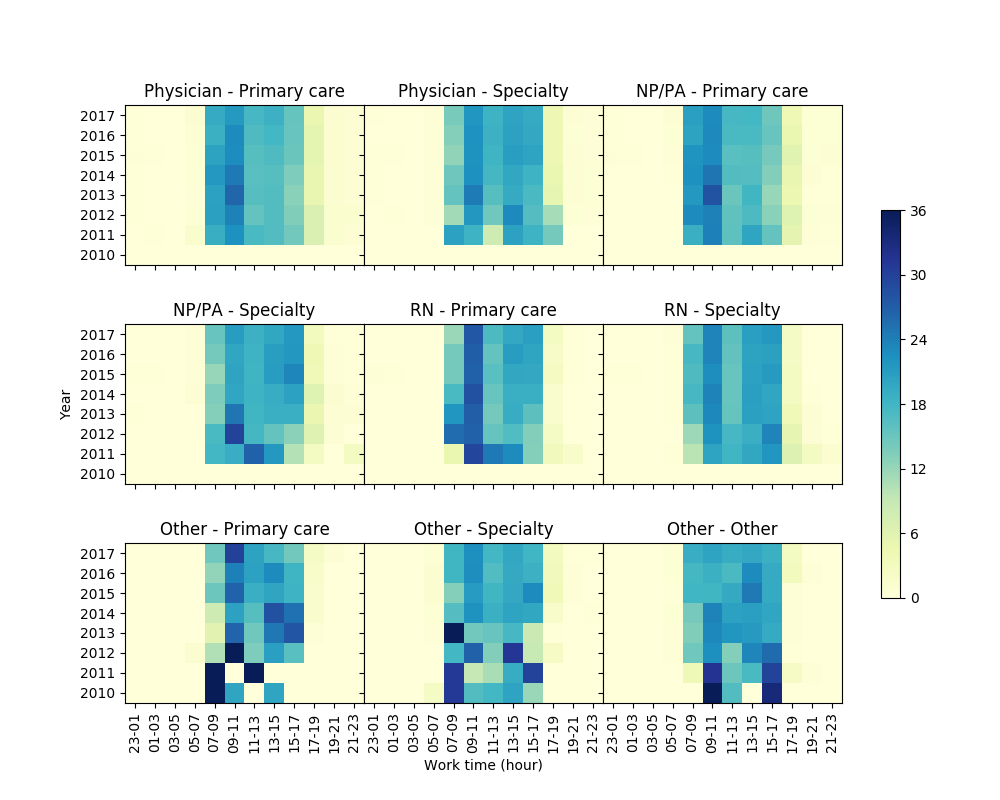


Figure S8 Time distribution (%) of clinician-initiated messages in a day


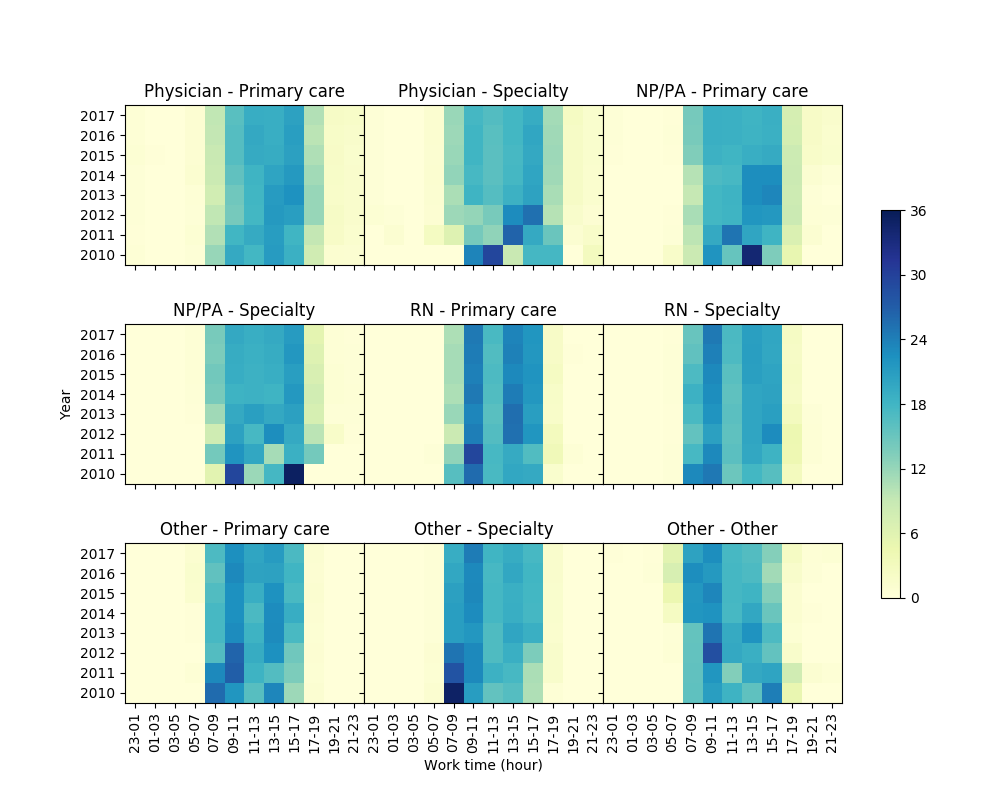


Figure S9 Time distribution (%) of clinician-replied messages in a day

1. **Thread-oriented analysis**

We identified 1,576,205 message threads in 3,887,542 messages, which include 1,332,931 patient-initiated messages and 243,274 clinician-initiated messages, for thread-oriented analysis. We observed that 236,241 patient-initiated messages did not receive clinician responses. As verification, we randomly selected 100 such patient messages and found for most of them, clinicians did not need to reply or chose to directly call the patients. For example, one patient messaged to check whether the clinician had received his test results and requested a follow-up phone call.

Table S4 Statistics of time delay in the clinician responses to patient messages and back-and-forth rounds in the message threads (message thread lengths)

|  | Minimum | Maximum | Mean | Median |
| --- | --- | --- | --- | --- |
| Time delay (days) | 1 second | 1577.96 | 1.23 | 0.56 |
| Message thread length | 2 | 34 | 2.47 | 2 |

The longest time a clinician spent in responding patients was 1577.96 days. After inspecting the clinician responses and patient messages with very long delays, we found that in this scenario, clinicians intended to message those specific patients, yet picked up a random, long-time-ago thread to reply to.
